# Cases of Cancrum Oris

**Published:** 1852-05

**Authors:** Charles Brackett

**Affiliations:** Rochester, Ind.


ART. III.—*Cases of Cancrum Oris.*

*Messrs. Editors*—THE following are the partial details of three cases of Cancrum Oris, which you may publish if you think them of sufficient interest.

CASE I.—— —- Brown, aged about two years, had been for the greater part of its life subject to frequent attacks of Intermittent. The father, cachectic for several years past with chronic enlargement of the spleen, and some symptoms of mesenteric disease, had been frequently salivated, the last time during the winter of '50—51, from which lesions were formed in the mucous membranes of the mouth and palate. He never recovered, but died soon after his child from splenitis with typhoid symptoms: an illustration of the evil effects of malaria, the injudicious use of calomel, and bad hygienic management.

The child was taken with ulcerated spots on the gums, insides of the lips and cheeks, the ulcers first shallow, irregular, of a yellow ash color, spreading gradually and hardening the tissues in their immediate vicinity. When I first saw it, November 1st, 1851, there was a black gangrenous spot occupying the larger part of the upper lip; thence it spread rapidly taking the cheeks, nose, and under lip in its course, together with the whole superior maxillary bone, which fell into the mouth the night of the child's death, having been completely detached from its articulations with the other bones.

CASE II.—J. K., aged about eleven, whose father died a short time previously of typhoid fever, supervening on dysentery, had at the same time had a similar attack from which he imperfectly recovered.

On Tuesday, November 11, '51, the boy complained of chillness alternately with fever, breath fetid, gums spongy, and ulcerated as was the inside of the cheeks. The general symptoms were typhoid. I put him on a modification of the saline treatment. On Wednesday, the 26th, he had so far improved that I left him for a few days; on my return, December 2d, I was much surprised to find him with gangrene, or rather sphacelus of both cheeks which were cold and black. The erosion continued steadily to progress till the 8th, when he died.

CASE III.—Was in Newark, ten miles east. It pursued a course similar to the above, and with a like termination. There were in this vicinity, the past fall, an unusual number of cases of this disease, of greater or less degrees of severity, but which as a general rule yielded readily to remedies.

The treatment most successful in my hands were horse-mint, (Monarda Punctata) nit. argt. and mur. tinct. fer. locally; cod liver oil, antacid and tonic laxatives. The two fatal cases appeared however to be in no way beneficially influenced by these remedies, or by iod. quin. acid muriatic, or any other remedies known to us. The boy who died in Newark received no remedial treatment, had never taken any of the mercurial preparations. Brown's child, as I learn, had taken calomel and blue mass repeatedly during its whole life. The boy Kitt, during my attendance on him took, Nov. 14, five grains of mass hydgr., this was all the mercurial he had from me, but during the past three years, the mother said he had repeatedly taken calomel, in fact "he had lived on it."

Now, how far these three fatal cases had their origin from the use of calomel, I cannot certainly say; but, of course, it is denounced as the sole cause.

It is well here to remark that the parents of both K. and B. were not accustomed to ventilate thoroughly their apartments, and that at K.'s at the time of the boy's sickness two others were ill of typhoid. Yours, &c., CHARLES BRACKETT, M. D.

ROCHESTER, Ind., April 1, 1852.

